# Identification and characterization of aptameric inhibitors of human neutrophil elastase

**DOI:** 10.1016/j.jbc.2023.104889

**Published:** 2023-06-05

**Authors:** Stanisław Malicki, Mirosław Książek, Alicja Sochaj Gregorczyk, Marta Kamińska, Anna Golda, Barbara Chruścicka, Danuta Mizgalska, Jan Potempa, Hans-Peter Marti, Joanna Kozieł, Maciej Wieczorek, Jerzy Pieczykolan, Piotr Mydel, Grzegorz Dubin

**Affiliations:** 1 Laboratory of Proteolysis and Post-translational Modification of Proteins, Malopolska Centre of Biotechnology, Jagiellonian University, Krakow, Poland; 2 Department of Microbiology, Faculty of Biochemistry, Biophysics and Biotechnology, Jagiellonian University, Krakow, Poland; 3 Broegelmann Research Laboratory, University of Bergen, Bergen, Norway; 4 Department of Oral Immunity and Infectious Diseases, University of Louisville School of Dentistry, Louisville, Kentucky, USA; 5 Department of Clinical Medicine, University of Bergen, Bergen, Norway; 6 Innovative Drugs R&D Department, Celon Pharma Inc, Lomianki, Poland; 7 Protein Crystallography Research, Group Malopolska Centre of Biotechnology, Jagiellonian University, Krakow, Poland

**Keywords:** human neutrophil elastase, neutrophil, elastase, aptamer, pulmonary disease

## Abstract

Human neutrophil elastase (HNE) plays a pivotal role in innate immunity, inflammation, and tissue remodeling. Aberrant proteolytic activity of HNE contributes to organ destruction in various chronic inflammatory diseases including emphysema, asthma, and cystic fibrosis. Therefore, elastase inhibitors could alleviate the progression of these disorders. Here, we used the systematic evolution of ligands by exponential enrichment to develop ssDNA aptamers that specifically target HNE. We determined the specificity of the designed inhibitors and their inhibitory efficacy against HNE using biochemical and *in vitro* methods, including an assay of neutrophil activity. Our aptamers inhibit the elastinolytic activity of HNE with nanomolar potency and are highly specific for HNE and do not target other tested human proteases. As such, this study provides lead compounds suitable for the evaluation of their tissue-protective potential in animal models.

Human neutrophil elastase (HNE) is a member of the chymotrypsin superfamily of serine proteinases and is expressed primarily by neutrophils. Its intracellular function involves the degradation of foreign microorganisms engulfed by neutrophils (*e.g.*, periplasmic proteins or proteins associated with the outer cell membrane of Gram-negative bacteria) (1). Additionally, HNE is involved in signaling and the physiological turnover of endogenous proteins. HNE activities include the activation of protease-activated receptor 2 (2), the degradation of extracellular matrix proteins (*e.g.*, elastin, collagen, fibronectin, and proteoglycans) (3, 4), the inactivation of endogenous proteinase inhibitors (1), the release of growth factors and proinflammatory cytokines (2, 5, 6, 7), and the cell migration (8).

Because of the capacity of HNE to degrade multiple components of the extracellular matrix, the activity of HNE is tightly regulated. Within neutrophils, HNE is spatially compartmentalized into azurophilic granules. Once HNE is released into the extracellular space, alpha-1-antitrypsin and serpin B1, which are serine protease inhibitors of the serpin superfamily, control the activity of HNE (6, 9, 10, 11).

HNE elastinolytic activity is implicated in the pathogenesis of several chronic inflammatory diseases, in which it contributes to irreversible structural damage of the organs. This phenomenon is observed especially during intense inflammatory responses during which the balance between the activity of proteolytic enzymes and their inhibitors is disrupted. Dysregulated activity of HNE is implicated in the pathogenesis of various acute and chronic inflammatory conditions of the lung including emphysema (12), asthma (13), cystic fibrosis (14), obstructive pulmonary disease (15), bronchiectasis (16), acute lung injury (17), and acute respiratory distress syndrome (18, 19), as well as the progression of lung cancer (20, 21, 22).

Numerous studies investigating the potential of elastase inhibition in inflammatory disease yielded encouraging results (19). However, the development of small-molecule inhibitors with high selectivity for the active site of the target protease has proved challenging. Inhibitors that have low HNE specificity are often associated with numerous deleterious side effects that exclude them from clinical use. Nucleic acid aptamers directed against proteases offer a novel type of high-specificity, high-affinity ligand that can be used for targeting not only proteolytic activity, but also exosite interactions that mediate other functions. Unlike small-molecule inhibitors, which most often bind to the active site of target enzymes, aptamers are characterized by multiple interactions with the surface residues of target proteins (23, 24, 25, 26, 27, 28). Since the surface residues do not take part in catalysis or binding of the substrate and therefore are highly variable and unique for an individual protein target, we explored the possibility of developing a highly specific aptamer-based HNE inhibitor. Recently published data (29, 30, 31, 32, 33), including ours (34), indicates the possibility of developing aptameric-based HNE inhibitors with K_i_ values in the nanomolar range. Aptamers described to date inhibit target proteases with K_i_ values of 2.1 nM to 2.5 μM (29, 30, 31, 32, 33, 34).

Herein, we describe the development of highly specific, DNA-based inhibitors of HNE. The efficacy of our novel inhibitors was evaluated *in vitro* and *ex vivo* using human peripheral blood neutrophils. Our results expand the pipeline of HNE-binding molecules with favorable pharmacologic properties. Future interventional studies are necessary to evaluate the safety and efficacy of the provided molecules *in vivo*, but our results constitute a first step in the development of a treatment that alleviates HNE-mediated damage in inflammatory syndromes.

## Results

### Selection of HNE-directed aptamers

**Figure 1:**
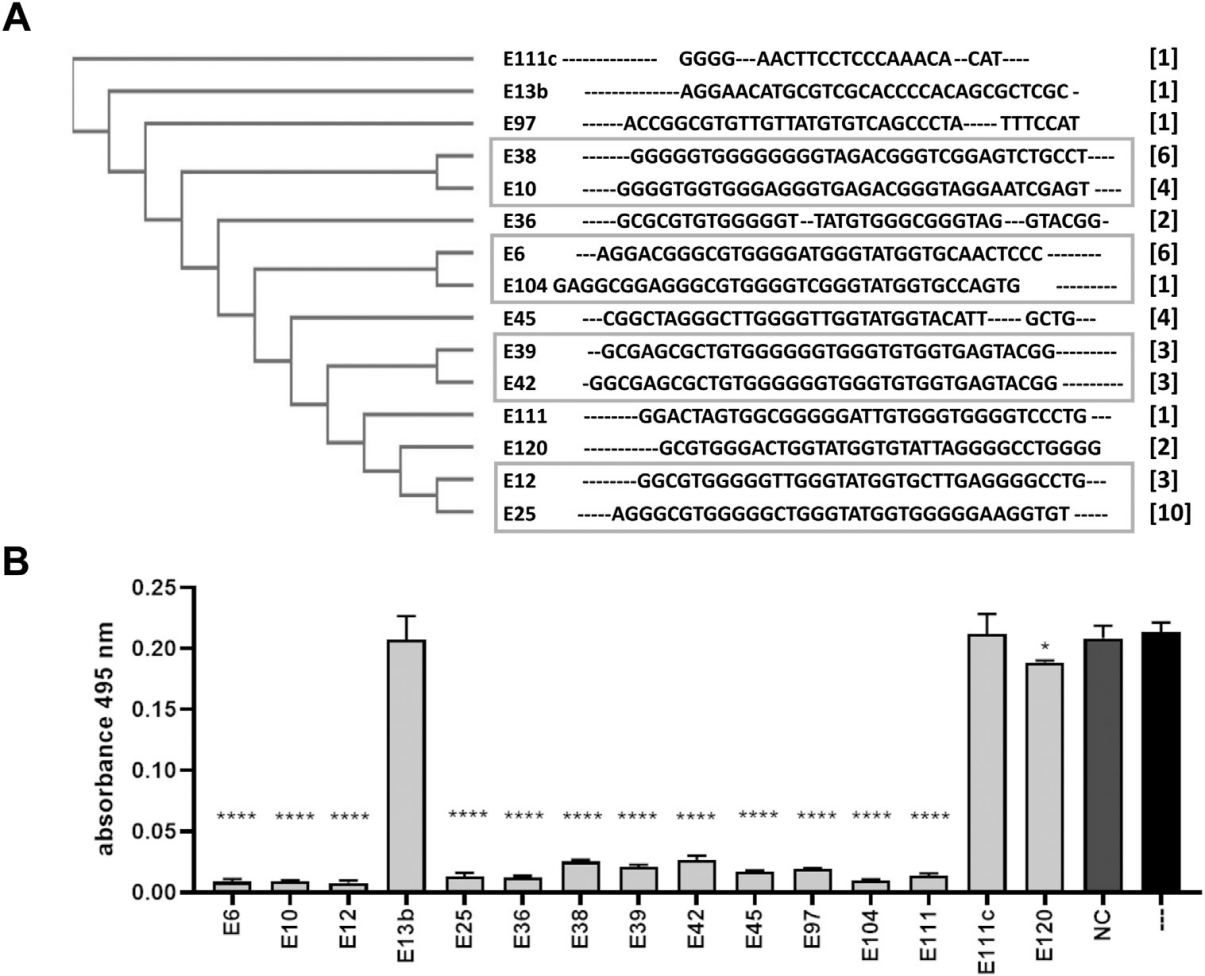
**Aptamer selection.***A*, the alignment and phylogenetic tree of sequences obtained from the last selection cycle. The phylogram was created using the Clustal Omega program (https://www.ebi.ac.uk/Tools/msa/clustalo/). The constant region of the library (primer attachment sites) is omitted from the analysis. The number of copies of a particular sequence in the overall pool (48 sequences) is shown on the *right* [in parentheses]; groups of highly homologous aptamers are *boxed*. *B*, HNE (100 nM) inhibition by representative aptamers (1.25 μM) determined using elastin–Congo red as a substrate. The graphs represent mean ± SD from three independent experiments (each performed in duplicate). Statistical significance (∗) was assessed using one-way ANOVA by comparing each result with a no-inhibitor control (∗∗∗∗*p* < 0.0001). HNE, human neutrophil elastase.

ssDNA aptamers targeting HNE were selected in three parallel systematic evolution of ligands by exponential enrichment (SELEX) procedures, as depicted schematically in Fig. S1*A*. Aptamer pools from selected enrichment cycles were screened for interactions with HNE by ELISA (Fig. S1*B*). Aptamer pools obtained from the final selection cycles were cloned and 120 randomly selected clones were sequenced (Fig. S2). Multiple sequence alignment revealed four groups of highly similar sequences and several unrelated sequences, some containing relatively few nucleotides (Fig. 1*A*). Interestingly, despite the varied selection conditions used in parallel SELEX cycles, each of the four identified homology clusters contained sequences derived from multiple SELEX procedures.

Sequences representing each of the identified clusters and an additional seven representing the non-clustering sequence pool were tested for their ability to inhibit HNE in elastin–Congo red assay. Twelve out of fifteen tested aptamers inhibited the activity of HNE (Fig. 1*B*). Three of the developed similar-length ssDNA sequences were inactive, demonstrating that HNE inhibition is sequence-specific. Four aptamers (E6, E10, E12, and E104) that had the highest inhibitory activity (coincidentally, each from a different sequence cluster) were selected for further evaluation.

### Initial characterization of HNE-targeting aptamers

**Figure 2:**
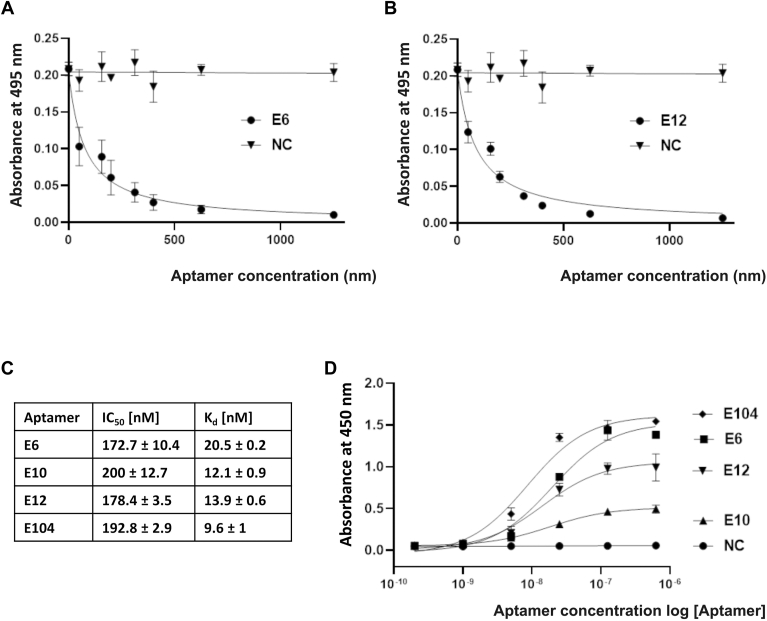
**The activity of the developed aptamers.***A* and *B*, concentration-dependent inhibition of HNE (100 nM) activity by the indicated aptamers using elastin–Congo red as the substrate. *C*, the IC_50_ values of the four developed inhibitors with the highest inhibitory activity and binding K_d_ (one site-specific binding model implemented in GraphPad Prism software was used to determine the K_d_ value based on ELISA datapoints). *D*, the concentration-dependent interaction between HNE and the indicated biotinylated aptamers using ELISA. HNE, human neutrophil elastase; NC, nonspecific sequence of ssDNA.

The HNE inhibitory potency of the selected aptamers was evaluated in an elastin–Congo red assay. The residual activity of HNE was monitored using elastin–Congo red as a substrate with increasing concentrations of tested aptamers (Figs. 2 and S3). In this assay, E6 and E12 aptamers had the lowest IC_50_ values (172.7 nM and 178.4 Nm, respectively). Random (nonspecific) ssDNA sequence (NC) of identical length that were selected as aptamers displayed no HNE inhibition potency even at the highest concentrations tested, demonstrating that inhibitory activity is sequence-specific.

The elastin–Congo red test allowed an estimate of the inhibitory activity of the tested aptamers. Since aptamers bind to surface amino acids and therefore do not necessarily cover the active site of the enzyme, we next evaluated the ability of the selected aptamers to bind to HNE using an ELISA assay (Fig. 2*D*). Aptamer E104 interacted most efficiently (K_d_ = 9.6 nM); however, strong binding did not correlate with inhibitory activity. E12 and E6 aptamers, showing a higher inhibitory potential (IC_50_ = 172.7 nM and 178.4 nM, respectively), compared to E104 (EC_50_ = 192,8 nM) were characterized by weaker binding (K_d_ = 20.5 nM and 13.9 nM for E6 and E12, respectively). The mechanistic explanation of this finding is provided in the discussion section. A nonspecific ssDNA sequence did not bind to HNE, indicating that the physical interaction between the aptamer and HNE is ssDNA sequence-specific. We selected the E6 and E12 aptamers (Table 1) for further analysis as these were the most active in the assays of inhibitory activity.

**Table 1:** The sequences of E6 and E12 aptamers

Aptamer E6	5′-**CATGCTTCCCCAGGGAGATGA**GGACGGGCGTGGGGATGGGTATGGTGCAACTCCC**GAGGAACATGCGTCGCAAAC**-3′
Aptamer E12	5′-**CATGCTTCCCCAGGGAGATG**GGCGTGGGGGTTGGGTATGGTGCTTGAGGGGCCTG**GAGGAACATGCGTCGCAAAC**-3′

The flanking sequences are shown in bold.

To predict the potential structure of selected aptamers, we analyzed the quadruplex content using software available at https://bioinformatics.ramapo.edu/QGRS/analyze.php. According to the analysis, aptamer E6 contains one potential quadruplex, while aptamer E12 contains two quadruplexes (Fig. S4*A*). Additionally, analysis using UNAFold (available at http://www.unafold.org/mfold/applications/dna-folding-form.php) suggests that E6 aptamer contains three hairpin loops, while the aptamer E12 contains two hairpin loops (Fig. S4*B*). The experimental analysis of quadruplex content was not attempted in this study.

### The size of protected substrates

**Figure 3:**
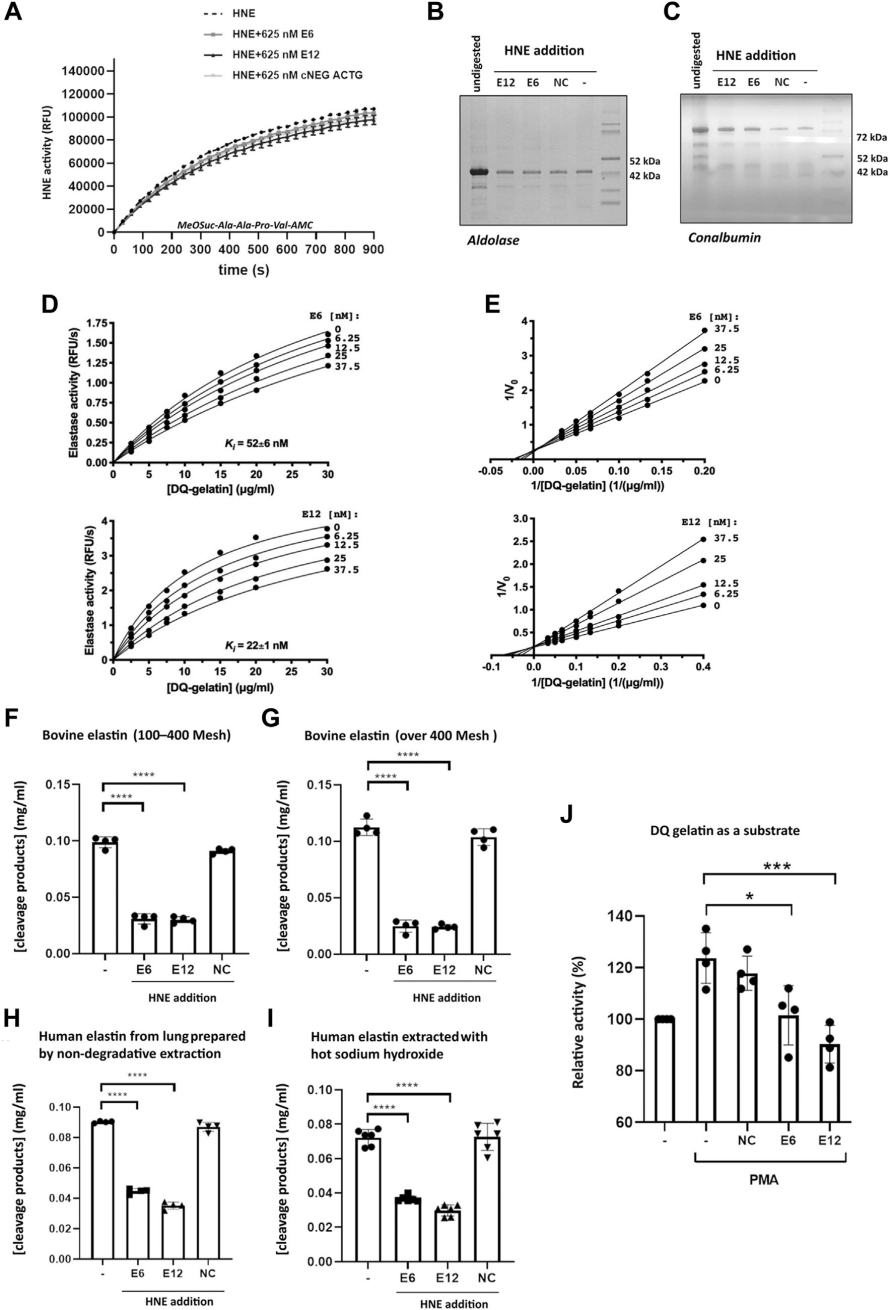
**The inhibitory activity of the tested aptamers depends on the molecular weight of the substrate.***A*, aptamers E6 and E12 had little effect on the HNE-mediated hydrolysis of a low-molecular-weight peptide substrate (MeOSuc-Ala-Ala-Pro-Val-AMC). *B* and *C*, aptamers E6 and E12 partially inhibited the degradation of conalbumin (75 kDa), but not that of aldolase (40 kDa) (the gel was cropped to the size of the analyzed proteins). Estimation of *K*_*i*_ values (*D*) and inhibition mode (*E*); Lineweaver–Burk plots of the aptamers using DQ gelatin as a substrate. Aptamers E6 and E12 inhibited elastinolytic activity of HNE. *F*, bovine elastin; particle size, 100 to 400 Mesh. *G*, bovine elastin; particle size, over 400 Mesh. *H*, human elastin extracted from adult lung (HS395). *I*, human elastin extracted from adult lung (HL457). *J*, aptamers E6 and E12 inhibited HNE activity in activated human neutrophils isolated from the peripheral blood of healthy donors. The graphs represent the mean ± SD of at least three independent experiments performed in duplicate. Statistical significance (∗) was assessed using one-way ANOVA. HNE, human neutrophil elastase; NC, scrambled-aptamer control; PMA, PKC-activating phorbol ester.

**Figure 4:**
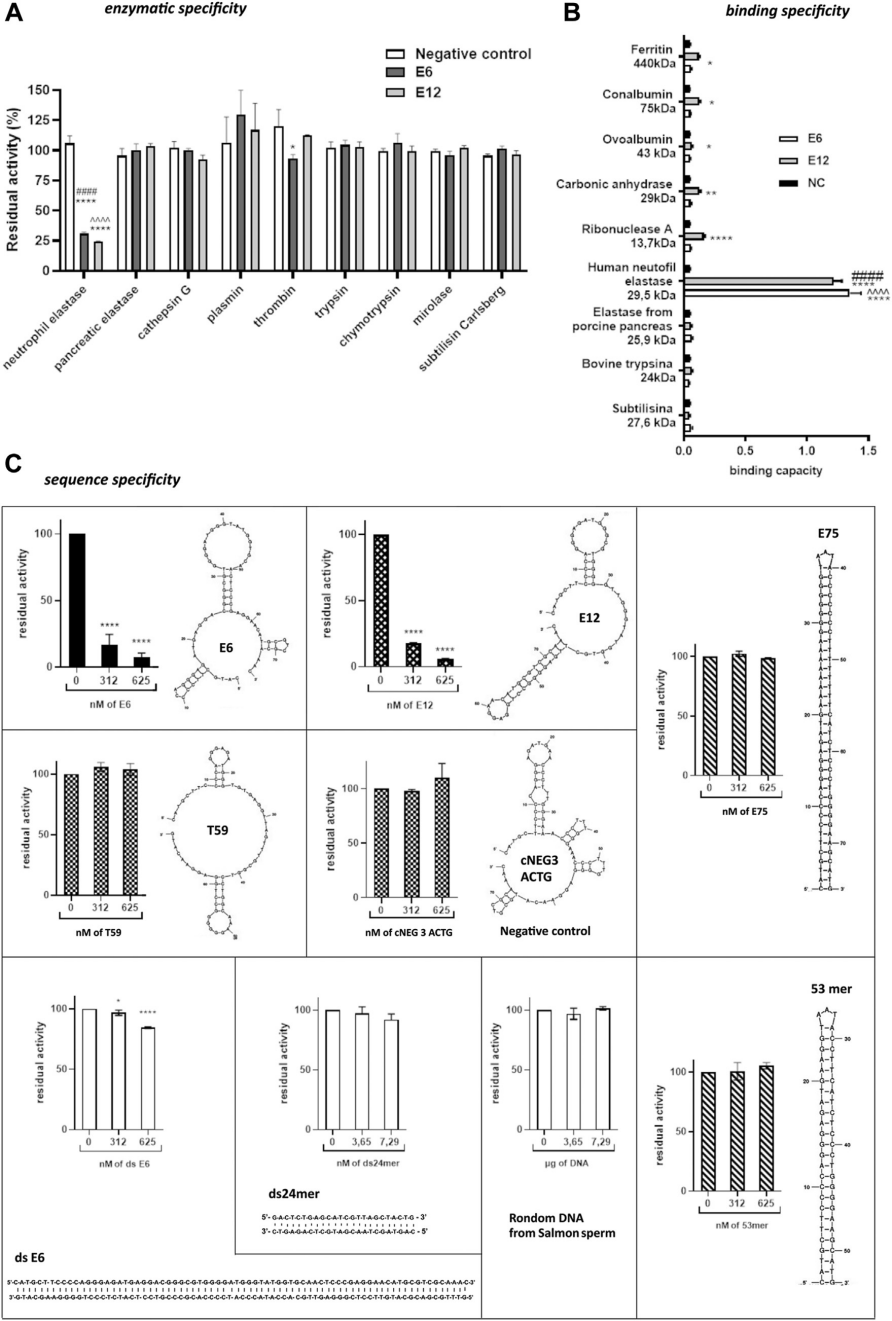
**The selectivity of E6 and E12.***A*, the aptamers inhibited the proteolytic activity of HNE, but not that of other tested proteases. *B*, the aptamers bound to HNE, but not to the other tested proteins. *C*, inhibition of HNE by nontarget aptamers and nonspecific DNA. The predicted secondary structures of the aptamers were calculated using MFold. Statistical significance was evaluated by two-way ANOVA (*panel A*, statistical significance differences in inhibition of various enzyme activities between aptamers E6 and E12 and the negative control sequence is denoted by ∗; compared with E6 inhibition of all other proteins is denoted by #; compared with E12 inhibition of all other proteins is denoted by ˆ; *panel B*, statistical significance compared with the negative control is denoted by ∗; compared with binding of E12 to all other proteins is denoted by #; compared with binding of E6 to all other protein is denoted by ˆ; *panel C*, statistical significance compared with uninhibited enzyme is denoted by ∗). HNE, human neutrophil elastase.

While determining the kinetic parameters that characterize aptamer inhibition, we encountered an apparent inconsistency in our results. The tested aptamers inhibited the hydrolysis of elastin but did not affect the hydrolysis of peptide substrates. Detailed analysis demonstrated that neither aptamer E6 nor E12 significantly inhibited HNE-mediated hydrolysis of MeOSuc-Ala-Ala-Ala-Val-AMC (Fig. 3*A*). In addition, these aptamers did not affect the hydrolysis of a small protein substrate, a 40 kDa monomer of aldolase 3. However, aptamers E6 and E12 inhibited the hydrolysis of a larger molecular weight protein substrate (conalbumin; 75 kDa). Hydrolysis of the large molecular weight, crosslinked substrates elastin, DQ-gelatin, and Azocoll (azo-dye impregnated collagen) was also inhibited by the aptamers (Figs. 3, *C*, *D*–*J* and 4*A*). These results allowed us to conclude that the aptamers most likely bind outside the substrate-binding pocket of elastase and sterically hinder the recognition of bulky substrates, but not small molecular weight substrates. The maximum molecular weight of a substrate able to access the active site of HNE in complex with an aptamer is 40 to 70 kDa.

In parallel to testing the aptamers against the routinely used elastin–Congo red substrate, we evaluated several other commercial preparations of elastin. Under standardized conditions, aptamers E6 and E12 inhibited the hydrolysis of elastin–Congo red (Fig. 2, *A* and *B*), native bovine elastin (regardless of its particle size), and native human elastin (Fig. 3, *F*–*I*). The levels of E6 and E12 inhibition of elastin hydrolysis varied slightly depending on the type of elastin; 90% for the dye-modified substrate, 70% for bovine elastin with small particle sizes below 37 μm, 80% for bovine elastin with large particle sizes (37–149 μm), 50% for human lung elastin by aptamer E6, and 60% for human lung elastin by aptamer E12. We attribute these apparent differences in inhibition to the purity of commercial elastin preparations, as we noted significant differences in the amounts of soluble protein released from elastin fibers during washing.

### Inhibition mode and K_i_ values

Aptamer-mediated inhibition of HNE followed Michaelis–Menten kinetics; the enzyme activity was dependent on the concentration of both the substrate and the inhibitor (Fig. 3*E*). The mode of inhibition was assessed by plotting the experimental data using the Lineweaver–Burk equation (Fig. 3*F*). The straight lines that were fitted to the experimental data for both tested aptamers (E6 and E12) intersected roughly at a single point located on the *Y*-axis, indicating that competitive inhibition occurred. Accordingly, the steady-state inhibition constant (K_i_) was determined to be the single parameter most suited for describing the potency of the inhibitors. The K_i_ value (*i*.*e*., the potency) of the E6 aptamer was 52 ± 6 nM, and the K_i_ value of the E12 aptamer was 22 ± 1 nM.

### The selectivity of HNE-targeted aptamers

To determine the selectivity of aptamers E6 and E12 toward HNE and exclude off-target inhibition of other proteases, we tested the activity of E6 and E12 against the following proteases: human cathepsin G (a neutrophil granule protease), plasmin and thrombin (proteases found in human plasma), porcine pancreatic elastase, bovine trypsin and chymotrypsin, subtilisin Carlsberg, and mirolase (from *Tannerella forsythia*). The E6 and E12 aptamers affected the activity of HNE only (Fig. 4*A*), demonstrating that both aptamers are selective.

The specificity of the E6 and E12 aptamers was tested in terms of their binding affinity to several non-HNE proteins (molecular weight 13–440 kDa) immobilized on a plastic surface as determined by ELISA (Fig. 4*B*). Aptamers E6 and E12 exhibited strong affinity to HNE, demonstrating that this assay format does not influence the aptamer conformation. Aptamer E6 did not bind to the other tested proteins. However, aptamer E12 was significantly less specific than E6 (E12 produced a statistically significant binding signal for several nontarget proteins compared with the negative control).

### Inhibition of HNE by aptamers E6 and E12 is sequence-specific

Several studies reported that HNE activity is nonspecifically inhibited by short DNA fragments (35, 36). The aptamers E13, E111c, E120, and NC had no effect on HNE elastinolytic activity (Fig. 1*B*), which suggests that the inhibition of specific HNE activities observed in our studies depends on the aptamer sequence and is not related to a general inhibitory effect of DNA fragments that was observed in earlier studies. Nevertheless, we tested this assumption more rigorously before further characterizing the aptamers.

To this end, we evaluated the effect on HNE of a number of semirandomly selected ssDNA and dsDNA fragments that contained the same number of nucleotides as our inhibitory aptamers. Nonspecific DNA fragments containing the flanking sequences of the original library, antitrypsin aptamer T59 (34), and the in-house developed single-stranded sequences forming hairpin structures (53mer and E75) did not significantly affect HNE activity when tested at concentrations at which both E6 and E12 inhibited HNE-mediated proteolysis of elastin–Congo red by more than 90% (Fig. 4*C*). Moreover, a randomly fragmented salmon sperm DNA sequence had no effect on HNE activity. A dsDNA 24-mer reported previously to inhibit elastase when tested using low-molecular-weight chromogenic substrates (36) did inhibit HNE activity in our assay, but the activity of HNE was reduced only by 10% (compared with 90% HNE inhibition by E6 and E12 at an equivalent concentration).

Pre-annealing of aptamer E6 with its antisense sequence reduced HNE activity by 16% compared with a 90% inhibition of HNE activity by single-stranded E6. This result demonstrates that the tertiary structure of the aptamer is responsible for inhibiting HNE. The residual inhibition mediated by double-stranded E6 is likely attributed to residual single-stranded E6 remaining in solution; however, this was not tested systematically.

Overall, these results demonstrate that the inhibition of HNE by the aptamers obtained in this study is specific to the aptamer sequence and is unrelated to a general effect of HNE inhibition by short DNA reported earlier.

### Aptamers E6 and E12 reduce the proteolytic potential of activated neutrophils

To determine the effect of the aptamers in cell culture conditions, we evaluated the effect of E6 and E12 on DQ gelatin degradation by activated neutrophils. Neutrophils were collected from the blood of healthy donors, treated with either E6 or E12 aptamers, a control aptamer, or untreated for 1 h, activated with PKC-activating phorbol ester (phorbol 12-myristate 13-acetate; PMA) for 3 h, and then the proteolytic activity in the cell supernatants was determined. As expected, PMA treatment significantly increased the proteolytic activity in the culture supernatant (Fig. 3*J*). A scrambled, negative control sequence only partially decreased the proteolytic activity of the supernatants. The E6 aptamer significantly decreased the proteolytic activity, and the E12 aptamer decreased the proteolytic activity to the level of that of the unstimulated neutrophil control. These results indicate that the E6 and E12 aptamers are active in a complex cell culture environment.

## Discussion

HNE plays a crucial role in the effective innate immune response against invading pathogens. At the same time, the prolonged uncontrolled activity of HNE results in pathological degradation of the extracellular matrix, which is particularly evident in diseases of the respiratory system. In this regard, HNE is an established drug target for cystic fibrosis, chronic obstructive pulmonary disease, and many other respiratory diseases (19). The therapeutic potential of elastase inhibition has been validated in many preclinical and clinical studies (19). Recent data indicate that HNE may be involved in coronavirus disease 2019 pathogenesis, since increased HNE levels at the site of infection enhance the activation of the spike protein, thus facilitating host cell entry of 614G severe acute respiratory syndrome coronavirus 2 (37). Moreover, prompt administration of HNE inhibitors may be helpful in patients with COVID-19 suffering from acute respiratory distress syndrome (38, 39).

HNE plays a key role in the destruction of the extracellular matrix. This study aimed to develop new highly specific aptamer inhibitors of HNE that could be used effectively to attenuate the destructive potential of this enzyme toward the extracellular matrix. High specificity is a unique and superior feature of aptamers compared with low-molecular-weight inhibitors, including peptides. Our DNA aptamers have high selectivity in terms of both binding to the target protein and inhibition of the enzyme activity. Based on our current study, we anticipate that HNE-targeted aptamers will have limited undesirable effects *in vivo*; however, this remains to be demonstrated.

The inhibition constants for aptamers E6 and E12 were 52 nM and 22 nM, respectively. Therefore, they are superior to most previously characterized oligonucleotide-based protease inhibitors. Other reported types of aptamers inhibit the activity of proteases with K_i_ values between 2.1 nM to 2.5 μM; the majority have K_i_ values of several dozen nanomoles (29, 30, 31, 32, 33, 34). Moreover, our aptamers have Ki values comparable to those of HNE inhibitors used in clinical trials, for example, ONO-5046 (sivelestat; trade name, Eraspol), which has a K_i_ value of 200 nM (40). Previous studies reported that DNA reduces the proteolytic activity of elastase against low-molecular-weight chromogenic substrates (36, 41) as well as its elastinolytic activity (35, 41). In our experiments, a very low concentration of randomly selected ssDNA or dsDNA fragments had a limited impact on the elastinolytic activity of HNE, whereas sequence-specific aptamers had a pronounced effect. This finding indicates that the nonspecific action of DNA described by others requires much higher concentrations and is probably based on the weak, nonspecific binding properties of DNA. The sequence-specific inhibition and the nonspecific inhibition likely rely on different mechanisms. Both E6 and E12 aptamers inhibit HNE activity against bulky substrates, but not small molecular weight substrates. Protease inhibition dependent on the molecular weight of the substrate is well described for human α2-macroglobulin (42, 43). Human α2-macroglobulin traps active proteases within its hollow interior, thus isolating the proteases from bulky substrates, while the small substrates may still diffuse into the hollow and be targeted by a protease. A comparable mechanism is unlikely for aptamers because of their relatively small molecular weight. Instead, we postulate that the aptamers described in this study bind in close vicinity to the active site, not close enough to interfere with the hydrolysis of small substrates, but sufficiently close to sterically hinder the access of bulky substrates. Through this binding mode, the aptamers would competitively inhibit the hydrolysis of bulky substrates, but not small substrates.

During the selection of aptamers, the E104 aptamer was identified as having better binding capacity to HNE than the E6 and E12 aptamers, but having a lower inhibitory potential. Presumably, this aptamer binds to HNE without significantly obstructing the active site, and thus the binding does not interfere with substrate recognition. The interpretation of our data indicates that the obtained aptamers bind to HNE at different sites. We anticipate that further functional and structural analysis of the aptamers will indicate that it will be beneficial to combine two or three aptamers with the best parameters, as was done previously with antithrombin and anti-vascular endothelial growth factor aptamers (44). The combination of aptamers will allow the construction of an inhibitor with a cage structure, which will have much better functional properties, and the aptamers within the structure will synergistically inhibit HNE. The aptamers obtained in this study are characterized by the presence of sequences potentially capable of forming quadruplexes. It is significant that other aptameric inhibitors described so far can also form quadruplexes (45), including one of the first developed and described aptamers, the human thrombin inhibitors (46, 47). The quadruplex structures show high structural stability (48), which is advantageous for maintaining biological activity.

In summary, we have developed highly specific and potent ssDNA aptamers that selectively target HNE and abolish its elastinolytic activity. The discovery of these aptamers provides an opportunity to limit HNE-mediated damage of the extracellular matrix and so relieve the symptoms of chronic respiratory tract diseases. The general nontoxic nature of aptamers and their target-protease specificity suggests that they will have fewer adverse effects than current protease inhibitors; however, further research is needed to establish their efficacy and any side effects *in vivo*.

## Experimental procedures

### *In vitro* selection

ssDNA fragments exhibiting affinity toward HNE were selected using previously developed procedures (34, 49). In brief, HNE (Preparatis Ltd) was immobilized on Dynabeads M270 Amine (Thermo Fisher Scientific) using suberic acid bis(3-sulfo-N-hydroxysuccinimide ester) sodium salt ((3); Sigma-Aldrich) or alternatively on M-280 tosyl activated beads (Invitrogen), according to the manufacturer’s instructions (detailed protocols are provided in Supporting materials).

ssDNA library, consisting of a randomized 35-nucleotide region (N_35_) flanked with 20-nucleotide constant sequences: 5′-CATGCTTCCCCAGGGAGATG-N_35_-GAGGAACATGCGTCGCAAAC-3′ was synthesized on a 0.2 μM scale and purified using HPLC (IBA Lifesciences). The library was formulated in selection buffer: PBS (pH 7.4) supplemented with 5 mM MgCl_2_, 10 mM KCl and 0.01% Tween 20, yeast tRNA (2 μg/ml) (Invitrogen) and bovine serum albumin (0.12 mg/ml) (BioShop Canada, Inc) or alternatively bovine casein (0.12 mg/ml) (Sigma-Aldrich).

Before each selection cycle, the library (4 nanomoles of the library in the initial round) was denatured for 5 min at 92 °C, cooled on ice, and brought to room temperature. For negative selection, the entire library was incubated with noncoated beads (3–40 μl), and the supernatant was collected for selection cycles. For selection, immobilized elastase was incubated with the library for 10 to 20 min at 25 °C with shaking (1000 rpm). The amount of elastase was gradually reduced in subsequent selection cycles: from 1 μl to 0.4 μl of beads with immobilized HNE.

The beads were washed 3 to 4 times with selection buffer. Following washing, the beads were suspended in 400 μl of PCR reaction mixture (0.5 mM dNTPs, 2.5 mM MgCl_2_, 5U Taq polymerase [Thermo Fisher Scientific]), unmodified forward primer: 5′-CATGCTTCCCCAGGGAGAGG-3' (0.625 μM) and modified reverse primer: 5' Phosphate-GTTTGCGACGCATGTTCCTC-3' (0.625 μM), subjected to initial denaturation step (95 °C, 5 min) and 35 polymerase cycles (30 s 95 °C; 30 s 53 °C; 30 s 72 °C), followed by a polishing cycle of 72 °C for 5 min. PCR products were extracted with phenol-chloroform- isoamyl alcohol and precipitated overnight with ethanol at −20 °C. The DNA pellet was recovered by centrifugation, washed with 70% ethanol, dried, and dissolved in dH_2_O. The nonproductive strand was digested using phage λ exonuclease (Thermo Fisher Scientific), and the resulting ssDNA library was directed to the subsequent selection cycle.

The selection strategy and library enrichment in elastase-binding sequences are summarized in Fig. S1. Detailed selection conditions are presented in accordance with the MAPS guideline proposed by McKeague *et al*. (50) in Tables S1 and S2.

### Postselection identification of aptamers

After completing the last selection cycle, the aptamer pool was PCR amplified using unmodified primers (35 polymerase cycles [30 s, 95 °C; 30 s, 53 °C; 30 s, 72 °C] followed by a polishing cycle of 72 °C for 7 min). The PCR products were ligated into the pTZ57R/T vector (InsTAclone PCR Cloning Kit, Fermentas), clones were obtained in *Escherichia coli* DH5α, and tested for the presence of desired inserts by colony PCR with M13 primers (M13fwd:5′-GTAAAACGACGGCCAGT-3′, M13rev:5′-CAGGAAACAGCTATGAC-3′). Each positive clone was sequenced across the aptamer coding region (Genomed S. A.). The resulting sequences were grouped into clusters using T-Coffe (http://www.ebi.ac.uk/Tools/msa/tcoffee/; Fig. S2). Single sequence representative of each cluster was selected (Fig. S2) for evaluation of HNE inhibition.

### Elastase interaction assay

Ninety-six–well plates (Nunc) were coated with HNE (10 μg/ml) (Athens Research & Technology, Inc) at 10 μg/ml in PBS. The plates were washed with selection buffer, and 100 μl of tested biotinylated aptamer was added in the same buffer (250 ng total pool in enrichment tests during selection; serial dilutions from 0 to 1.25 μM for comparative analysis of affinity). 5′-biotin CATGCTTCCCCAGGGAGATGAACCCTTTGGGAAACCCTTTGGGAAACCCTTTGGGGAGGAACATGCGTCGCAAAC was used to control for nonspecific binding. After 20 min incubation, unbound aptamers were removed by washing. Streptavidin-horseradish peroxidase (R&D Systems, Inc) was added (according to the manufacturer's procedure) for detection, and after 20 min, the unbound reagent was removed by washing. A hundred microliters of horseradish peroxidase substrate (R&D Systems, Inc) was added and incubated for 5 min when the reaction was stopped by the addition of 50 μl of 1 N H_2_SO_4_. Absorbance was determined at 450 nm using the Infinite 200 PRO multimode reader (Tecan Group Ltd).

To analyze the specificity of aptamers in interaction with proteins, the plates were coated at 10 μg/ml with subtilisin, bovine trypsin, porcine pancreatic elastase, ribonuclease A, carbonic anhydrase, ovalbumin, conalbumin, and ferritin. Apparent affinity was determined exactly as for HNE.

### HNE inhibition

(Detailed protocols are provided in Supporting materials).

HNE was contacted for 15 min with tested aptamers in assay buffer (50 mM Tris, pH 7.5 containing 150 mM NaCl, 5 mM CaCl_2_, 10 mM KCl, 5 mM MgCl_2_, and 0.02% Tween 20) at 37 °C. The residual activity was monitored at 37 °C using the following substrates and detection methods.

*MeOSuc-Ala-Ala-Pro-Val-AMC* (1.5 mM; Merck) was incubated with 25 nM HNE and 0 to 5 μM aptamer. The released fluorescence was monitored at Ex/Em = 380/460 nm.

*Elastin Congo-red* (Sigma-Aldrich), *human elastin* from lungs (HL457 and HS395), and *bovine elastin* from neck ligament (ES60 and E60) (all from Elastin Products Company, Inc) in suspension at 2.5 mg/ml, were incubated with 100 nM HNE and 0 to 1.25 μM aptamer. Following 24 h incubation at 37 °C, the undigested substrate was removed by centrifugation (5 min, 15,000 rpm). The reaction product was determined in the supernatant by absorbance at 495 nm for Elastin Congo-red or by the Bradford method for unmodified elastin.

*Aldolase* (5 μM) and *conalbumin* (2.67 μM) (both from GE Healthcare) were incubated with 2 ng/μl (68 nM) elastase and 625 nM aptamer. The reaction products (24 h, 37 °C) were analyzed by SDS-PAGE.

### Inhibition mode and *K*_*i*_

HNE (5 nM) was incubated with increasing concentrations of aptamers E6 and E12 (0–37.5 nM) in the assay buffer (100 μl) for 15 min at 37 °C. An equal volume of DQ-gelatin (5–60 μg/ml) was added, and the reaction progress was monitored as fluorescence increase (Ex/Em = 495/515 nm). The mode of inhibition was determined graphically using the Lineweaver-Burk plot and Equation 1:

(1) $$\frac{1}{V}=\frac{K_{m}}{V_{\max}}\times \frac{1}{\left[S\right]}+\frac{1}{V_{\max}}$$

where V is the reaction velocity, V_max_ is the maximum V, K_m_ is the Michaelis–Menten constant, and [S] is the substrate concentration.

The inhibition constant *K*_*i*_ was determined assuming competitive inhibition, using GraphPad Prism (https://www.graphpad.com) and Equation 2:

(2) $$V=V_{\max}\times \frac{\left[S\right]}{K_{m}^{obs}+\left[S\right]};\;K_{m}^{obs}=K_{m}\times \left(1+\frac{\left[I\right]}{K_{i}}\right)$$

where $K_{m}^{obs}$ is the Michaelis–Menten constant in the presence of inhibitor, [I] is inhibitor concentration, and *K*_*i*_ is the inhibition constant.

### Specificity of protease inhibition

HNE, human plasmin, thrombin and cathepsin G, porcine pancreatic elastase, bovine trypsin and chymotrypsin, mirolase (*T. forsythia*), and subtilisin Carlsberg (*Bacillus subtilis*), each at 0.5 μM concentration were incubated for 15 min at 37 °C with 10-fold molar excess of random sequence aptamer, E6 and E12 in 0.1 M Tris, pH 7.5 containing 150 mM NaCl, 5 mM CaCl_2_, 10 mM KCl, 5 mM MgCl_2_, 0.05% Tween-20, and 0.02% NaN_3_. Azocoll (Calbiochem) suspension was added to a final concentration of 5 mg/ml, and the samples were incubated with shaking at 37 °C for 1 h. The undigested substrate was removed by centrifugation (5 min, 16,000 rcf), and the extent of hydrolysis was determined as absorbance of the supernatant at 410 nm.

### Inhibition of proteolytic activity of human peripheral blood neutrophils

Human peripheral blood was obtained from a blood bank in accordance with relevant legal regulations. Neutrophils were isolated from granulocyte-enriched fraction by gradient centrifugation as described before (51). In brief, 3 ml Ficoll-Paque (Cytiva) was added to 4 ml of the blood, and the sample was centrifuged at 400*g* for 40 min at 20 °C. Mononuclear cells were harvested and neutrophils were isolated by centrifugation (280*g*, 10 min) after 30 min incubation with 1% polyvinyl alcohol. Neutrophils were collected from the upper layer. Residual erythrocytes were removed by lysis in water. Neutrophils were resuspended in serum-free Dulbecco's modified Eagle's medium without phenol red (Thermo Fisher Scientific) and seeded at 0.5 × 10^6^/well on poly-L-lysine (0.01 mg/ml; Sigma-Aldrich) coated 96-well plates. The plates were spun down (200*g*, 5 min) to allow cells to adhere to the plates. The cells were incubated with tested aptamers (125 μM) for 60 min, followed by stimulation with 25 nM phorbol ester (PMA; Sigma-Aldrich) for 3 h at 37 °C. The supernatants were collected, and proteolytic activity was assayed. Hundred microliters of DQ-gelatin (20 μg/ml) in 0.2 M Tris, pH 7.5 containing 300 mM NaCl, 20 mM KCl, 10 mM MgCl_2_, 0.05% Tween-20, and 0.02% NaN_3_ was added to 100 μl of tested supernatant, and the progress of the reaction was monitored as fluorescence increase at Ex/Em = 495/515.

### Statistical analysis

Data were analyzed using GraphPad Prism (GraphPad Software Inc). Significance of observed differences in binding potency and inhibitory activity between tested aptamers and the control was analyzed using one-way ANOVA. Specificity of binding and inhibition of potential targets was analyzed using two-way ANOVA with follow-up Tukey’s multiple comparison test. All data are presented as mean ± SD. Differences between groups were considered significant for *p* < 0.05, where ∗*p* < 0.05, ∗∗*p* < 0.01, ∗∗∗*p* < 0.001, and ∗∗∗∗*p* < 0.0001. The numbers of independent experiments are specified in relevant figure legends.

## Data availability

The data presented in this study are available in the main text and the Supporting materials of this article.

## Supporting information

This article contains Supporting information.

## Conflict of interest

The authors declare that they have no conflicts of interest with the contents of this article.
