# Supplementary material for: Identification and characterization of aptameric inhibitors of human neutrophil elastase
Source: J Biol Chem. 2023 Jun 5;299(8):104889. doi: 10.1016/j.jbc.2023.104889 (PMC10359491; doi:10.1016/j.jbc.2023.104889)
Supplement: Supporting information [file mmc1.pdf]

**Identification and characterization of aptameric inhibitors of human neutrophil elastase - supporting information**

Stanisław Malicki, Mirosław Książek, Alicja Sochaj Gregorczyk, Marta Kamińska, Anna Golda, Barbara Chruścicka, Danuta Mizgalska, Jan Potempa, Hans-Peter Marti, Joanna Kozieł, Maciej Wieczorek, Jerzy Pieczykolan, Piotr Mydel and Grzegorz Dubin

**Supporting information**

Experimental Procedures:
HNE immobilization on M270 amine Dynabeads
HNE immobilization on Dynabeads™ M-280 Tosylactivated
Digestion dsDNA with λ exonuclease
Removal of non-specific products of the PCR reaction
HNE inhibition by aptamers

Tables:
Table 1. Selection conditions.
Table 2. Selection conditions for each round of a new SELEX experiment

Figures:
Figure S1 A summary of the process used to select HNE-binding aptamers.
Figure S2 The sequences of the pools of aptamers obtained from SELEX.
Figure S3 The aptamer concentration-dependently inhibited the hydrolysis of elastin–Congo red (2.5 mg/ml) by HNE (100 nM).
Figure S4  Sequence analysis of E6 and E12 aptamers.

**HNE immobilization on M270 amine Dynabeads**

Immobilization of the protein on the M270 amine beads was carried out using the chemical linker: suberic acid bis(3-sulfo-N-hydroxysuccinimide ester) sodium salt (BS3) (method developed and optimized in-house). To immobilize the target protein, 10 µl of M270 amine beads were washed three times with 0.5 ml of 0.1M phosphate buffer (pH 7.4) supplemented with 0.15M NaCl and then suspended in 10 µl of this buffer. Six µl of 100mM BS3 was added, gently mixed and incubated for 30 min in room temperature. Linker excess was removed by washing the beads three times in the phosphate buffer with 0.15 M NaCl. The beads were resuspended in 10 µl of phosphate buffer and 2 µl of human neutrophil elastase (at a concentration 10 mg/ml at 18U/mg) was added. Beads were mixed gently. After 45 min of incubation in RT the supernatant was removed, and 500 µl of 0.05M Tris-HCl (pH 7) was added. Beads were then incubated for a further 15 min at room temperature. Beads were rinsed three times with PBS buffer supplemented with 0.5% bovine albumin (BSA) and 0.1% Tween-20. During the selection process, in order to exclude aptamers capable of binding BSA from the selection, the beads with immobilized protein were also rinsed with casein buffer. The level of

immobilization was tested with the substrate: MeoSuc-AAPV-pNA (2mM) against the reagent blank (Beads free of HNE).

**HNE immobilization on Dynabeads™ M-280 Tosylactivated**

Hundred and sixty-five µl of Dynabeads™ M-280 Tosylactivated were washed twice with 1ml of 0.1 M Na-phosphate buffer, pH 7.4. A hundred ug of HNE was dissolved in 150 µl of phosphate buffer (0.1 M Na-phosphate buffer, pH 7.4) and added to the prepared beads. Then 100 µl of 3 M ammonium sulphate in 0.1 M Na-phosphate buffer, pH 7.4 was added. The sample was mixed gently by pipetting and incubated with gentle mixing for 16 hours in RT. The supernatant was then removed and 1 ml of PBS buffer, pH 7.4 with 0.5%(w/v) BSA was added to the beads. The beads were incubated in RT for 1 hour with gentle mixing. After this time, the supernatant was removed and the beads were washed twice with 1 ml of PBS buffer, pH 7.4 with 0.1% (w/v) BSA. The beads with the immobilized HNE were suspended in 100 µl PBS, pH 7.4 with 0.1% (w/v) BSA. The level of immobilization was tested with the substrate: MeOSuc-Ala-Ala-Pro-Val-pNA (2mM) against the reagent blank.

**Digestion dsDNA with λ exonuclease**

After each selection cycle, the PCR products were subjected to the phosphorylated strand digestion with lambda phage exonuclease (ThermoScientific) to recover the unmodified strand (single-stranded DNA pool). Digestion was performed for 1.5 h at 37 °C, with gentle shaking, in 500 µl mixture containing 100 U of λ exonuclease. After this time, the digests (ssDNA) were extracted with a mixture of phenol-chloroform-isoamyl alcohol, precipitated with EtOH and suspended in 100 µl d$H_2O$. The ssDNA samples were stored at -20°C before the next selection cycle.

**Removal of non-specific products of the PCR reaction**

During the selection process, an increasing amount of the non-specific PCR products, that did not correspond to the length of the 75 nucleotide library, was observed. To remove them, the samples were electrophoresed in 10% polyacrylamide gels with 7 M urea in 0.5 x TBE buffer for 90 min at 90 V at 4°C. The gel band corresponding to the 75-nucleotide long fragment was cut with a scalpel, repeatedly frozen (in liquid nitrogen) and thawed (at 37°C), and then desalted by incubation on patch filters placed on distilled water. The purified fragments were amplified by PCR. Finally, the DNA was extracted with a mixture of phenol, chloroform and isoamyl alcohol and precipitated with ethanol. The ssDNA samples were stored at -20°C before the next selection cycle.

**HNE inhibition by aptamers**

The inhibitory potential of aptamers against HNE was tested using: MeoSuc-AAPV-pNA (Sigma-Aldrich, St.Louis, Missouri, USA), FITC-casein (Thermo Fisher Scientific, Waltham, Massachusetts, USA) and elastin-Congo Red (Sigma-Aldrich, St. Louis, Missouri, USA) and unmodified proteins: human lung elastin prepared by non-degradative extraction of the adult lung (Elastin products company, Inc., Owensville, Missouri, USA), human lung elastin prepared by extraction of adult lung with hot sodium hydroxide (Elastin products company, Inc., Owensville, Missouri, USA). Elastin purified from bovine neck ligament (ES60 - Particle Size: Pass 400 Mesh and E60 - Particle Size: 100 - 400 Mesh), prepared by neutral extraction

method (Elastin Products Company, Inc., Owensville, Missouri, USA), conalbumin and aldolase, both from Gel Filtration Cal Kit (GE Healthcare, Chicago, Illinois, USA). The experiments were carried out in Elastase Aptamer Assay Buffer (EAAB) (50 mM Tris, 150 mM NaCl, 5 mM, 10 mM KCl, 5 mM $MgCl_2$, 0.02% Tween 20, pH 7.5).

*MeOSuc-Ala-Ala-Pro-Val-AMC*

Human neutrophil elastase in EAAB buffer was incubated for 15 min with aptamers in a black microtiter plate. The reaction was initiated by the addition of fluorogenic substrate MeO-Suc-Ala-Ala-Pro-Val-AMC (Merck, Darmstadt, Germany) to the final concentration of 1.5 mM with elastase at the final concentration of 25 nM and aptamer concentrations in the range of 0 - 5 μM. The activity was monitored for 40 minutes at 37°C (exc/em=380/460 nm) for 30 min, at 37°C employing SpectraMax GeminiEM reader (Molecular Devices, San Jose, California, USA).

*Elastin Congo-Red*

Human neutrophil elastase in EAAB buffer was incubated for 15 min with aptamers. The reaction was initiated by the addition of elastin-Congo Red to the final concentration of 2.5 mg/ml elastin with elastase at the final concentration of 3 ng/μl and aptamer concentrations in the range of 0-1.25 μM. Following 24 hour-incubation at 37°C with shaking, the undigested substrate was separated by centrifugation (5 min, 15000 rpm) and the absorbance of the supernatant was measured at 495 nm using the Infinite 200 PRO multimode reader (Tecan Group Ltd., Männedorf, Switzerland).

*Human and bovine elastin*

The bovine elastin purified from bovine neck ligament (ES60 - Particle Size: Pass 400 Mesh (Smaller than 37 μm), (E60 - Particle Size: 100 - 400 mesh (149-37 μm), HL457- Human Lung Elastin, prepared by extraction of the adult lung with hot sodium hydroxide, Human, HS395, Human Lung Elastin Prepared by non-degradative extraction of the adult lung) from Elastin Products Company, Inc. USA were used in the analysis.

Human neutrophil elastase in EAAB, was incubated for 15 min with aptamers. Following elastase inhibition, 250 μl of elastin suspension was added to the final concentration of 2.5 mg/ml, elastase 3 ng/μl, and aptamers 0 - 1.25 μM. Substrate digestion reactions were carried out for 3 - 24 hours at 37°C on a shaker. Undigested elastin was separated by centrifugation (5 min, 15000 rpm) and the concentration of the reaction product with the Bradford method was measured using the Infinite 200 PRO multimode reader (Tecan Group Ltd., Männedorf, Switzerland).

*Konalbumin, Aldolase*

5 μg Aldolase or 5 μg Conalbumin (GE Healthcare, Chicago, Illinois, USA) was digested with 2 ng/μl elastase in 25 μl EAAB alone or in presence of aptamers (625 nM) for 24 hours at 37°C, then separated using 10% SDS-PAGE. The digestion levels were analyzed by Coomassie Brilliant Blue R-250 staining.

**Table S1. Selection conditions.**

| Selection condition | |
|---|---|
| Library information | • Length of the random region: 35 nucleotides; primer binding sites: forward - 20 nucleotides and reverse - 20 nucleotides<br>• Nucleic acid modifications: a modified reverse primer: 5' Phosphate (to facilitate the digestion of the reverse strand of dsDNA by exonuclease)<br>• Concentration of starting library: library stock: 100 µM, ssDNA concentration at the first selection cycle: 2 µM<br>• Library synthesis: ssDNA library was synthesized on a 0.2 µM scale and purified via HPLC (IBA Lifesciences, Göttingen, Germany) (Not amplified by PCR prior to Round 1) |
| Folding conditions | • Temperature and time for each step: before each selection cycle the library was denatured for 5 minutes at 92°C, cooled on ice (10 min), and brought to room temperature. All selection cycles were performed at 24°C or 37°C on the shaker for 10-20 min. |
| Buffer and pH | • Selection buffer: PBS (pH 7.4) supplemented with 5 mM $MgCl_2$, 10 mM KCl and 0.01% Tween 20 (used to prepare the Dynabeads with target protein and to separate poorly-bound aptamers during selection)<br>• Binding buffer: Selection buffer supplemented with: yeast tRNA and BSA, or alternatively bovine casein as specified below. Used during the binding and incubation of the aptamer pool with the protein immobilized on the bed.<br>Buffers were made immediately prior to use. |
| Additional constituents of binding/ selection buffer | Blocking agents or competitors: yeast tRNA (2 µg/ml; Invitrogen, Waltham, Massachusetts, USA) and BSA (0.12 mg/ml; BioShop Canada, Inc., Burlington, Ontario, Canada) or bovine casein (0.12 mg/ml; Sigma-Aldrich, St. Louis, Missouri, USA). |
| Immobilization of target | • Target protein - HNE (Preparatis Ltd, Krakow, Poland) was immobilized on Dynabeads M270 Amine (Thermo Fisher Scientific, Waltham, Massachusetts, USA) using as linker: suberic acid bis(3-sulfo-N-hydroxysuccinimide ester) sodium salt (BS3; Sigma-Aldrich, St. Louis, Missouri, USA) or alternatively on Dynabeads M-280 tosylactivated beads (Invitrogen, Waltham, Massachusetts, USA)<br>• Immobilization was confirmed through HNE activity detection using substrate: MeOSuc-Ala-Ala-Pro-Val-pNA |
| Partitioning conditions | • Aptamers were allowed to bind to the target protein for 10-20 min in the Binding buffer on a shaker at 24°C or 37°C, unbound aptamers were washed away with selection buffer (as specified in Table 2). |
| Negative/counter selection | • Negative selection:<br>The negative selection was performed before selection for HNE-specific aptamers. The negative selection was carried out for 20 min, at 24°C on a shaker - the supernatant was transferred to the beds with the target protein. The negative selection was performed for:<br>*a)* beds with linker - without target protein before 1 (20µl of beads), 2 (20µl), 3 (4ul), 4 (20µl), 5 (20µl), 6 (40µl), 7(33µl), , 10 (30µl), 11P (5µl), 3P (5µl) selection cycles.<br>*b)* beds with immobilized bovine albumin before 8 (30µl of beads), 9 (40ul of beads) cycle of selection.<br>c) beds with immobilized bovine casein before 7P (3µl of beads), 15P (3µl of beads), 6P* (3µl of beads) |

| | |
|---|---|
| | (bovine albumin immobilization and bovine albumin immobilization - protocol analogous to the target protein was used) |
| Preparation of Pool for each round | • single-stranded oligo generation:<br>– The beads with target protein and aptamers were suspended in the PCR reaction mixture and amplified in the PCR reaction.<br>– Products of the reaction (dsDNA) were extracted with phenol-chloroform isoamyl alcohol and precipitated overnight with ethanol.<br>– The non-productive strand was digested using phage λ exonuclease (Thermo Fisher Scientific, Waltham, Massachusetts, USA) then again extracted with phenol-chloroform isoamyl alcohol and precipitated overnight with ethanol at - 20°C and the resulting single-stranded DNA library was directed to subsequent selection cycle.<br>• quantification of the library after each round:<br>The DNA was analyzed by urea-denaturing gel separation and evaluated by spectrophotometry.<br>• oligonucleotide purification:<br>PCR products were extracted with phenol-chloroform isoamyl alcohol and precipitated overnight with ethanol at - 20°C. The DNA pellet was recovered by centrifugation, washed with 70% ethanol, dried and dissolved in $dH_2O$ |
| PCR conditions | • Polymerase: Taq polymerase (Thermo Fisher Scientific, Waltham, Massachusetts, USA) was used to amplify the DNA after each cycle.<br>Taq buffer: 100 mM Tris-HCl (pH 8.8 at 25°C), 500 mM KCl, 0.8% (v/v) Nonidet P40, supplemented with 2,5mM $MgCl_2$<br>• Primers: forward primer: 5'-CATGCTTCCCCAGGGAGAGG-3' (final conc. 0.625 µM) and modified reverse primer: 5' Phosphate - GTTTGCGACGCATGTTCCTC-3' (final conc. 0.625 µM)<br>• PCR cycle parameters: initial denaturation step (95°C, 5 min) and 35 polymerase cycles (30 sec. 95°C; 30 sec. 53°C; 30 sec. 72°C) followed by a final elongation step of 72°C for 5 minutes<br>• number of cycles in each selection step: 25-35<br>• Amplified samples were analyzed by electrophoretic separation in Urea-PAGE. |

**Table S2.** Selection conditions for each round of a new SELEX experiment

| **Round** | **Concentration of library/ amount of target used** | **Volume of binding buffer** | **Temperature and length of incubation** | **Number and length of washes** | **Number of PCR cycles to amplify bound species** |
|---|---|---|---|---|---|
| Round 1 | mol: 4 nmol, (volume: 40µl of 100µM synthesized libraries)/ | 2000 µl | 24 °C, 15 min. | 3x1ml (1 mins Each) | 25 cycles |

| | | | | | |
|---|---|---|---|---|---|
| | 1 µl of M270 Amine beads with immobilized HNE | | | | |
| Round 2 | 1µl of M270 Amine 0.5 nmol of ssDNA | 1000 µl | 24 °C and 15 min. | 3x1ml (1 mins Each) | 25 cycles |
| Round 3 | 1µl of M270 Amine/ 0.2 nmol of ssDNA | 1000 µl | 37 °C and 15 min. | 3x0,5ml (1mins Each) | 30 cycles |
| Round 4 | 1 µl beads M-280/ 0.2 nmol of ssDNA | 1000 µl | 24 °C, 15 min. | 4x1ml (1 mins Each) | 25 cycles |
| Round 5 | 1 µl beads M-280/ 0.2 nmol of ssDNA | 1000 µl | 24 °C, 15 min. | 3x1ml (1 mins Each) | 35 cycles |
| Round 6 | 0.5 µl beads M-280/ 0.2 nmol of ssDNA | 1000 µl | 24 °C, 15 min. | 3x1ml (1 mins Each) | 25 cycles |
| Round 7 | 0.165 µl beads M-280/ 0.2 nmol of ssDNA | 1000 µl | 24 °C, 15 min. | 3x1ml (1 mins Each) | 25 cycles |
| Round 8 | 0.5 µl beads M-280/ 0.4 nmol of ssDNA | 1000 µl | 24 °C, 10 min. | 3x1ml (1 mins Each) | 25 cycles |
| Round 9 | 0.165 µl beads M-280/ 0.4 nmol of ssDNA | 1000 µl | 24 °C and 20 min. | 3x1ml (1 mins Each) | 30 cycles |
| Round 10 | 0.165 µl beads M-280/ 0.4 nmol of ssDNA | 1000 µl | 24 °C and 20 min. | 3x1ml (1 mins Each) | 30 cycles |
| Round 11P | 0.5 µl of M270 Amine/ 0.09 nmol ssDNA | 1000 µl | 37 °C and 15 min. | 3x0.5ml (1mins Each) | 30 cycles |
| Round 12P | 0.5 µl of M270 Amine 0.18 nmol of ssDNA | 1000 µl | 24 °C and 15 min. | 3x0.5ml (1mins Each) | 30 cycles |
| Round 13P | 0.4 µl of M270 Amine/ 0.18 nmol of ssDNA | 1000 µl | 24 °C and 15 min. | 3x0.5ml (1mins Each) | 30 cycles |
| Round 14P | 0.4 µl of M270 Amine/ 0.18nmol of ssDNA | 1000 µl | 24 °C and 15 min. | 3x0.5ml (1mins Each) | 30 cycles |
| Round 15P | 0.4 µl of M270 Amine/ 0.09 nmol of ssDNA | 1000 µl | 37 °C and 10 min. | 3x0.5ml (1mins Each) | 30 cycles |
| Round 3P | 0.5 µl of M270 Amine/ 0.02nmols of ssDNA | 1000 µl | 24 °C and 15 min. | 3x0.5ml (1mins Each) | 30 cycles |
| Round 4P | 0.5 µl of M270 Amine/ 0.09 nmol of ssDNA | 1000 µl | 24 °C and 15 min. | 3x0.5ml (1mins Each) | 30 cycles |
| Round 5P | 0.4 µl of M270 Amine/ 0.18 nmol of ssDNA | 1000 µl | 24 °C and 15 min. | 3x0.5ml (1mins Each) | 30 cycles |
| Round 6P | 0.4 µl of M270 Amine/ 0.09nmols of ssDNA | 1000 µl | 24 °C and 15 min. | 3x0.5ml (1mins Each) | 30 cycles |
| Round 7P | 0.4 µl of M270 Amine/ 0.09nmol of ssDNA | 1000 µl | 37 °C and 10 min. | 3x0.5ml (1mins Each) | 30 cycles |
| Round 5P* | 0.5 µl of M270 Amine/ 0.18 nmol of ssDNA | 1000 µl | 24 °C and 15 min. | 3x0.5ml (1mins Each) | 30 cycles |
| Round 6P* | 0.4 µl of M270 Amine/ 0.09 nmol of ssDNA | 1000 µl | 37 °C and 10 min. | 3x0.5ml (1mins Each) | 30 cycles |

## Supporting figures

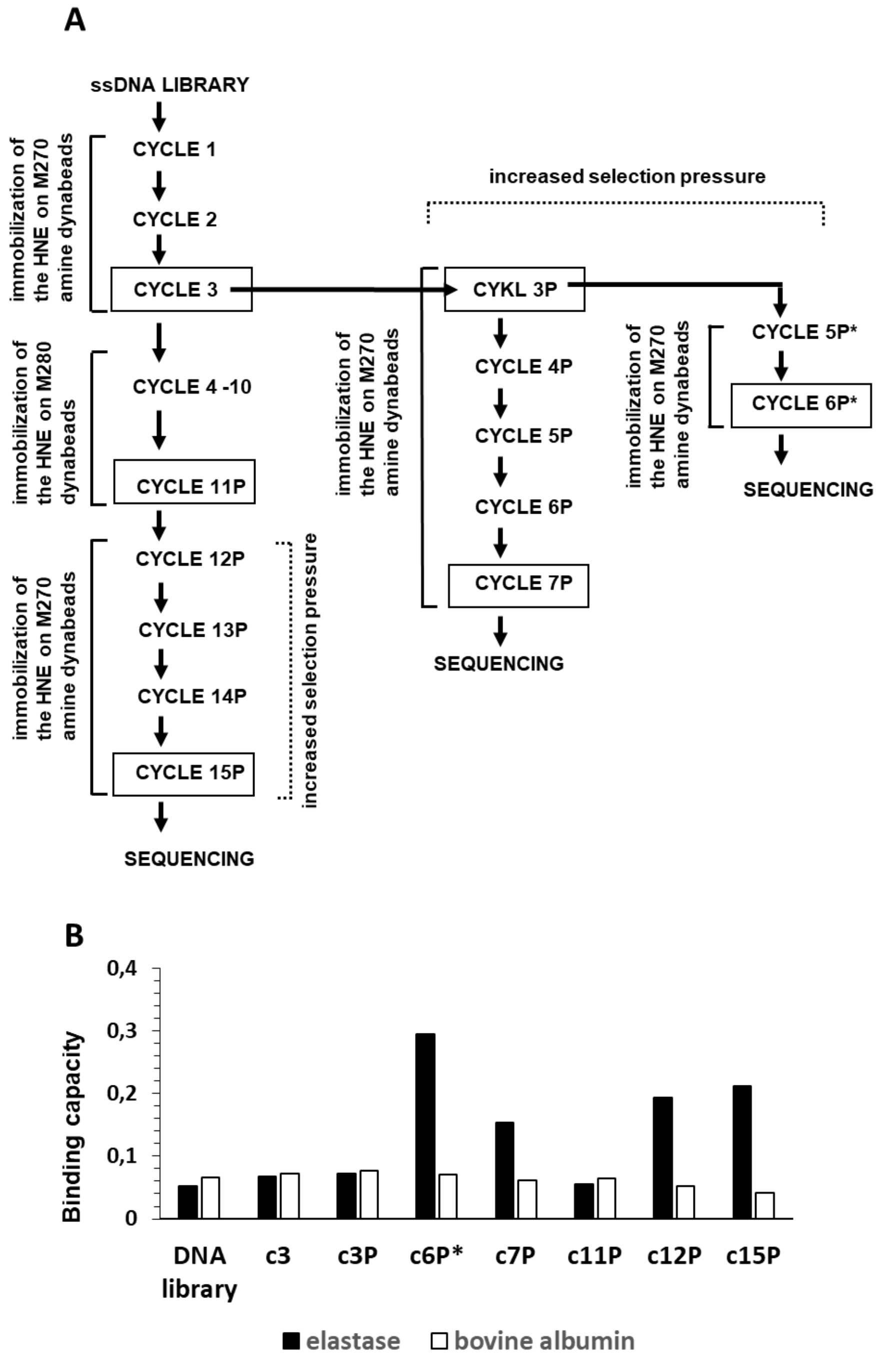


**Figure S1** A summary of the process used to select HNE-binding aptamers. (A) A schematic representation of the selection process, indicating the immobilization chemistry. The suffix “P” indicates cycles of increased selection pressure (low concentration of the reagents). An asterisk (*) indicates cycles where the ssDNA library required polyacrylamide gel purification to limit the amplification of products that had molecular weights significantly different from the initial library. Black rectangles indicate cycles tested in panel B. (B) The affinity of aptamer pools

taken at the selection cycles indicated in panel A toward the target protein (HNE) and the cross-reactivity with bovine albumin.

| | | |
|---|---|---|
| **Apt13b** | **------------AGGAACATGCGTCGCACCCCACAGC--GCTCGC------** | **31** |
| **Apt97c** | **-----------ACCGGCGTGTTGTTAT---GTGTC--AGCCCTATTTCCAT-** | **35** |
| **Apt111c** | **----------------------------GGGGAACTTCCTCCCAAACACAT-** | **23** |
| **Apt38** | **--GGGGGTGGGGGGGGTAGACGGGTCG--------------GAGTCTGCCT-** | **35** |
| Apt68 | -GGGGGGTGGGGGGGGTAGACGGGTCG--------------GAGTCTGCCT- | 36 |
| Apt74 | -GGGGGGTGGGGGGGGTAGACGGGTCG--------------GAGTCTGCCT- | 36 |
| Apt78c | --GGGGGTGGGGGGGGTAGACGGGTCG--------------GAGTCTGCCT- | 35 |
| Apt83 | --GGGGGTGGGGGGGGTAGACGGGTCG--------------GAGTCTGCCT- | 35 |
| Apt80 | ---GGGGGTGGGGGGGTAGACGGGTCG--------------GAGTCTGCCT- | 34 |
| **Apt10** | **GGGGTGGTGGGAGGGTGAGACGGGTAG--------------GAATCGAGT--** | **36** |
| Apt13 | GGGGTGGTGGGAGGGTGAGACGGGTAG--------------GAATCGAGT-- | 36 |
| Apt43 | GGGGTGGTGGGAGGGTGAGACGGGTAG--------------GAATCGAGT-- | 36 |
| Apt100 | --GGGGGTGGGAGGGATAGACGGGTAG--------------GAGTCGAGATG | 36 |
| **Apt36** | **------GCGCGTGTGGGG----GTTATGTGGGCGGGTAGGTACGG-------** | **35** |
| Apt62bshort | ----CGCTGCATGTGGGG----GTTGT--TGGGTGGGTGGTACGG------- | 35 |
| **Apt104** | **-GAGGCGGAGGGCGTGGGGTCGGGTATGGTGCCAGTG---------------** | **36** |
| **Apt45** | **----CGGCTAGGGCTTGGGGTTGGTATGGTACATTGCTG-------------** | **35** |
| Apt28 | ----CGGCTAGGGCTTGGGGTTGGTATGGTACATTGCTG------------- | 35 |
| Apt111d | ----CGGCTAGGGCTTGGGGTTGGTATGGTACATTGCTG------------- | 35 |
| Apt119 | ----CGGCTAGGGCTTGGGGTTGGTATGGTACATTGCTG------------- | 35 |
| **Apt6** | **----AGGACGGGCGTGGGGATGGGTATGGTGCAACTCCC-------------** | **35** |
| Apt46 | ----AGGACGGGCGTGGGGATGGGTATGGTGCAACTCCC------------- | 35 |
| Apt50b | ---GAGGACGGGCGTGGGGATGGGTATGGTGCAACTCCC------------- | 36 |
| Apt120c | ----AGGACGGGCGTGGGGATGGGTATGGTGCAACTCCC------------- | 35 |
| Apt59 | ----AGGTTTGGC-GTGGGGTTGGTATGGTGCATCTATCT------------ | 35 |
| Apt93 | -------GGGGGGTGGTAGCTGGCGATGGGGGGACGATTTCGC--------- | 36 |
| **Apt39** | **---GCGAGCGCTGTGGGGGGTGGGTGTGGTGAGTACGG--------------** | **35** |
| Apt66 | ---GCGAGCGCTGTGGGGGGTGGGTGTGGTGAGTACGG-------------- | 35 |
| Apt71 | ---GCGAGCGCTGTGGGGGGTGGGTGTGGTGAGTACGG-------------- | 35 |
| **Apt42** | **--GGCGAGCGCTGTGGGGGGTGGGTGTGGTGAGTACGG--------------** | **36** |
| Apt89 | --GGCGAGCGCTGTGGGGGGTGGGTGTGGTGAGTACGG-------------- | 36 |
| Apt95 | --GGCGAGCGCTGTGGGGGGTGGGTGTGGTGAGTACGGGAGCCAGGGAGAT- | 49 |
| **Apt111** | **---------GGACTAGTGGCGGGGGATTGTGGGTGGGGTCCCTG--------** | **35** |
| **Apt120** | **------------GCGTGGGACTGGTATGGTGTATTAGGGGCCTGGGG-----** | **35** |
| Apt118b | ------------GCGTGGGGCTGGTATGGTGTATTAGGGGACTGGGG----- | 35 |
| **Apt12a** | **---------GGCGTGGGGGTTGGGTATGGTGCTTGAGGGGCCTG--------** | **35** |
| Apt12 | ---------GGCGTGGGGGTTGGGTATGGTGCTTGAGGGGCCTG-------- | 35 |
| Apt77 | ---------GGCGTGGGGGTTGGGTATGGTGGGGGAAGTGATTG-------- | 35 |
| Apt112 | ------GAGGGCGTGGGGGCTGGGTATGGTGGGGGAAGGTGT---------- | 36 |
| **Apt25** | **-------AGGGCGTGGGGGCTGGGTATGGTGGGGGAAGGTGT----------** | **35** |
| Apt33 | -------AGGGCGTGGGGGCTGGGTATGGTGGGGGAAGGTGT---------- | 35 |
| Apt40 | -------AGGGCGTGGGGGCTGGGTATGGTGGGGGAAGGTGT---------- | 35 |
| Apt51 | -------AGGGCGTGGGGGCTGGGTATGGTGGGGGAAGGTGT---------- | 35 |
| Apt52 | -------AGGGCGTGGGGGCTGGGTATGGTGGGGGAAGGTGT---------- | 45 |
| Apt55 | -------AGGGCGTGGGGGCTGGGTATGGTGGGGGAAGGTGT---------- | 35 |
| Apt107b | -------AGGGCGTGGGGGCTGGGTATGGTGGGGGAAGGTGT---------- | 35 |
| Apt119b | -------AGGGCGTGGGGGCTGGGTATGGTGGGGGAAGGTGT---------- | 35 |
| Apt116 | -------AGGGGGAGGGGGCGGGGTAGGGTGGGGGAAGGTGT---------- | 35 |

**Figure S2** The sequences of the pools of aptamers obtained from SELEX. Aptamers analysed further in this study are marked bold.

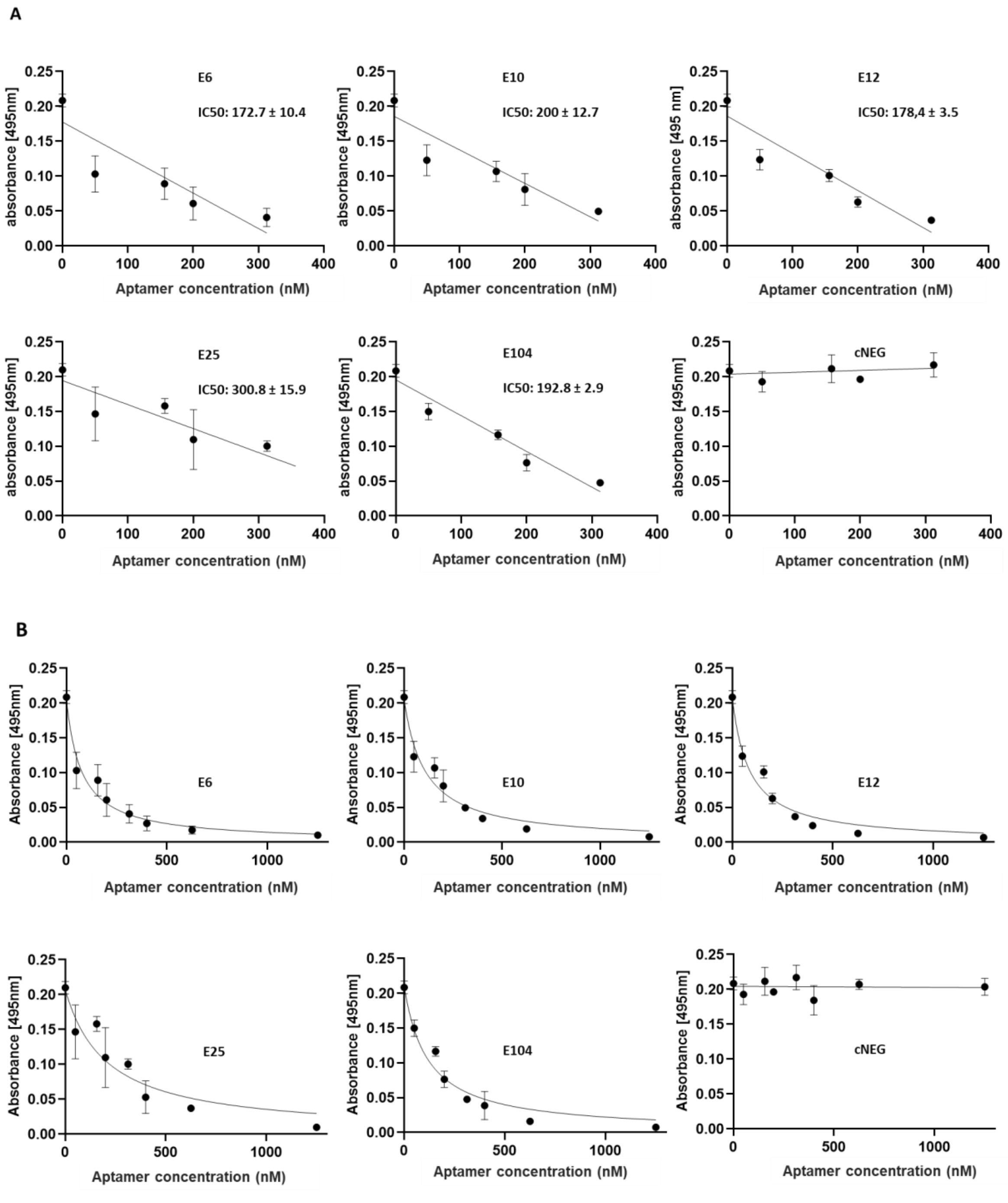


**Figure S3** The aptamer concentration-dependently inhibited the hydrolysis of elastin–Congo red (2.5 mg/ml) by HNE (100 nM). Data from the five aptamers with the highest inhibitory potential and non-specific sequence are shown ($IC_{50}$ ± SD). Linear (A) and nonlinear (B) regression analysis of the data.

**A**

E6

CATGCTTCCC CA**GGG**AGATG AGGAC**GGG**CG T**GGG**GAT**GGG** TATGGTGCAA CTCCCGAGGA ACATGCGTCG CAAAC

E12

CATGCTTCCC CA**GGG**AGAT**G GG**CGTG**GGG**G TT**GGG**TATGG TGCTTGA**GGG G**CCT**GG**A**GG**A ACATGCGTCG CAAAC

**B** E6 E12

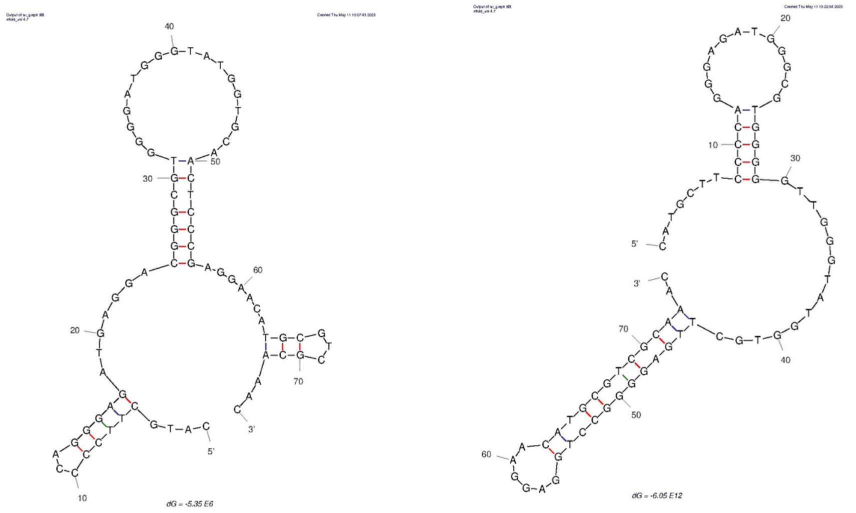


**Figure S4.** Sequence analysis of E6 and E12 aptamers. (A) Analysis of the presence of quadruplexes using software available at bioinformatics.ramapo.edu/QGRS/analyze.php (B) Secondary structure prediction using unafold.org/mfold/applications/dna- folding-form.php.
